# Laparoscopic surgery for an esophageal duplication cyst using a near‐infrared indocyanine green fluorescence system: A case report

**DOI:** 10.1111/ases.12729

**Published:** 2019-07-01

**Authors:** Mikito Mori, Kiyohiko Shuto, Atsushi Hirano, Chihiro Kosugi, Kazuo Narushima, Keiji Koda

**Affiliations:** ^1^ Department of Surgery Teikyo University Chiba Medical Center Ichihara Japan

**Keywords:** esophageal duplication cyst, laparoscopic surgery, NIR‐ICG fluorescence

## Abstract

We herein describe a case of laparoscopic surgery for an esophageal duplication cyst using a near‐infrared indocyanine green fluorescence system. A 64‐year‐old woman with a cystic tumor adjacent to the esophagogastric junction was referred to our hospital for treatment. Esophagogastroduodenoscopy and abdominal CT revealed a 70‐mm submucosal tumor derived from the abdominal esophagus. We performed laparoscopic resection and then evaluated the tissue perfusion of the abdominal esophagus by using a near‐infrared indocyanine green fluorescence system. A Dor fundoplication was performed to prevent postoperative gastroesophageal reflux disease and reinforce the mucosal layer defect. The postoperative course was uneventful, and pathological evaluation confirmed that the tumor was an esophageal duplication cyst. The patient did not develop recurrence in the 24 months after surgery. We have demonstrated that laparoscopic resection of an esophageal duplication cyst may be performed effectively with intraoperative assessment of tissue perfusion using a near‐infrared indocyanine green fluorescence system.

## 1 INTRODUCTION

Esophageal duplication cysts (EDCs) are rare congenital lesions, accounting for 0.5% to 2.5% of all esophageal tumors.^1^ It has been suggested that EDCs are caused by either incomplete embryologic recanalization (coalescence of vacuoles) or atypical blastogenesis of the primitive foregut, which usually occurs in the fifth to eighth week of gestation.^1^, ^2^ Most EDCs are localized in the distal thoracic esophagus, but some cases of intra‐abdominal EDCs have been reported. Several treatments are available for EDCs, including thoracotomy, thoracoscopic surgery, and endoscopic submucosal dissection.^3^, ^4^, ^5^ A few reports have described patients who underwent laparoscopic surgery for abdominal EDCs with excellent results.^6^ However, there have been no reports of laparoscopic resection of EDCs using a near‐infrared indocyanine green (NIR‐ICG) fluorescence system. We herein present a rare case of laparoscopic surgery for an EDC using an NIR‐ICG fluorescence system.

## 2 CASE PRESENTATION

**Figure 1:**
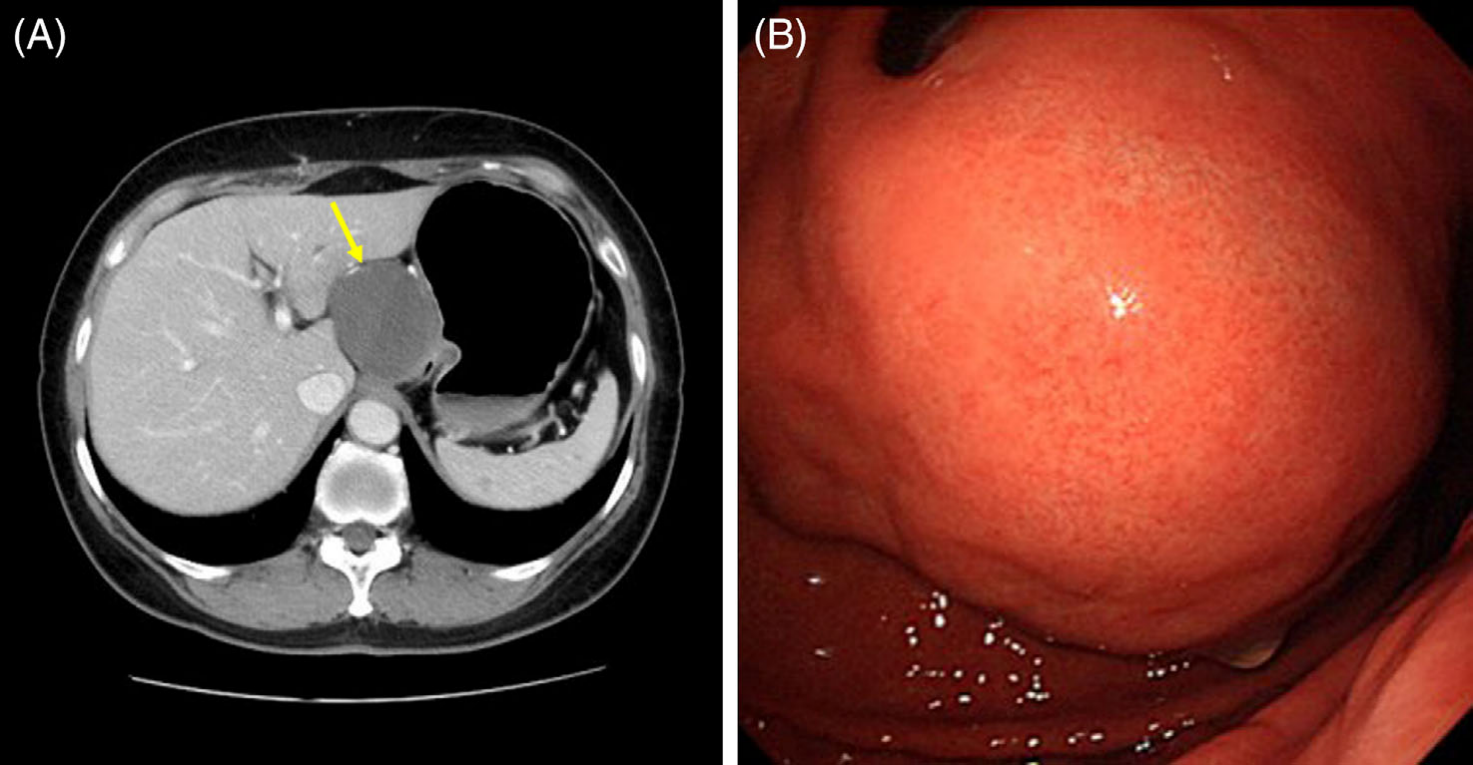
A, Esophagogastroduodenoscopic and B, abdominal CT images of an abdominal esophageal submucosal tumor. A 70‐mm submucosal tumor without mucosal defects in the abdominal esophagus was observed. The yellow arrow indicates the submucosal tumor in the abdominal esophagus

A 64‐year‐old woman with a cystic tumor near the esophagogastric junction was referred to our hospital for treatment. Esophagogastroduodenoscopy revealed a submucosal tumor with no mucosal defects in the abdominal esophagus adjacent to the esophagogastric junction. Abdominal CT showed a 70‐mm low‐density area in the abdominal esophagus without distant metastasis (Figure 1A,B). Thus, the patient was diagnosed with an abdominal esophageal submucosal tumor. The patient was thoroughly informed about several treatment options for the abdominal esophageal submucosal tumor and decided to undergo laparoscopic resection.

**Figure 2:**
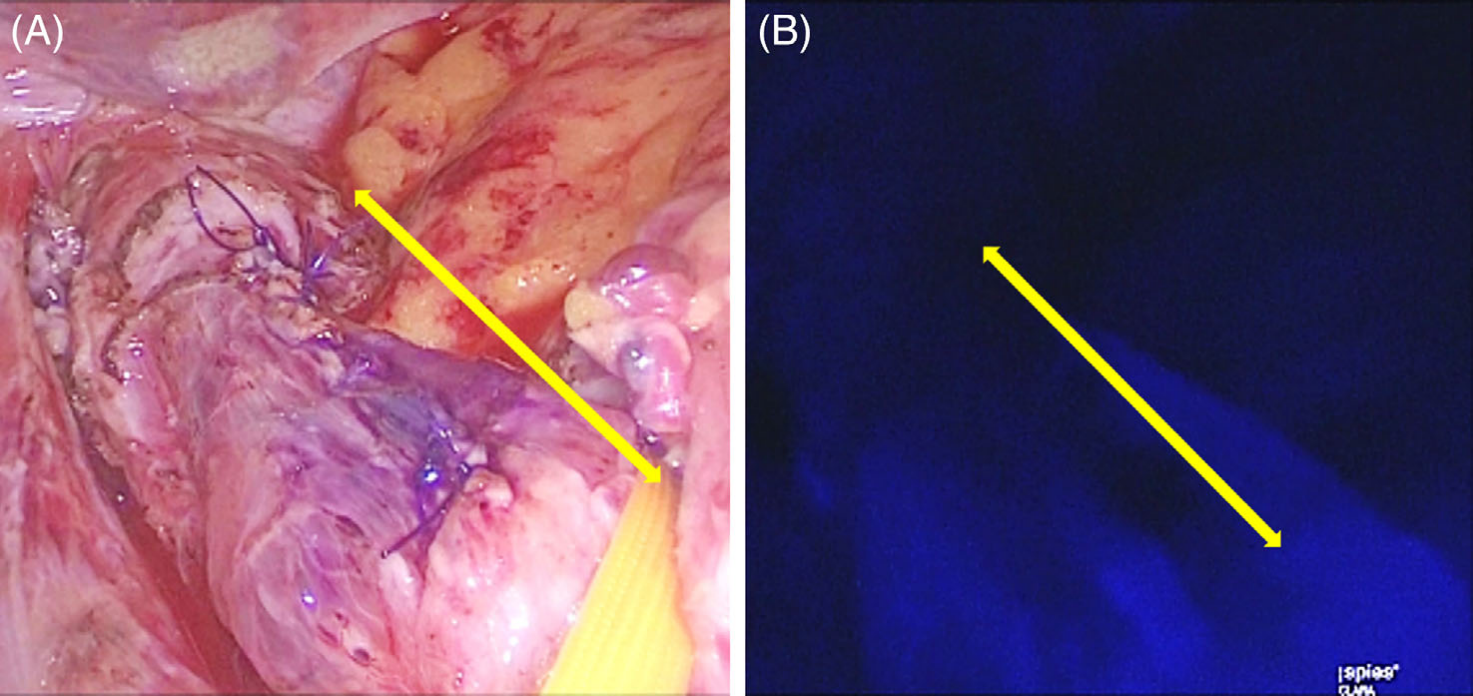
Intraoperative images of the excised lesion of the esophagus by A, white light and B, near‐infrared indocyanine green fluorescence. The yellow arrow indicates the dimension of the excised lesion of the esophagus

A camera port was inserted through an umbilical incision using an open technique. Under pneumoperitoneum of 10 mm Hg, four ports (two 5‐mm ports and two 12‐mm ports) were inserted into the right upper, right lower, left upper, and left lower quadrants, respectively. Esophageal adventitia layer dissection was followed by muscular layer dissection along the tumor without injury. After enucleation, tissue perfusion of the excised lesion in the abdominal esophagus was evaluated using an NIR‐ICG fluorescence system (IMAGE1 SPIES; Karl Storz, Tuttlingen, Germany), and hypoperfusion was found. A Dor fundoplication was performed to prevent postoperative gastroesophageal reflux disease (GERD) and reinforce the mucosal layer defect (Figure 2A,B). The operative time was 343 minutes, and the estimated blood loss was 10 mL. The patient was discharged on postoperative day 9 without adverse events.

Histopathological analysis revealed that the cyst wall was composed of two smooth muscle layers and was lined by pseudostratified ciliated columnar epithelium; the pathological diagnosis was an EDC. The patient was still alive with no recurrence or complications 24 months after surgery.

## 3 DISCUSSION

Esophageal duplication cysts are rare congenital anomalies that require surgical intervention. They account for approximately 10% to 15% of all gastrointestinal duplication cysts, and they are usually located in the lower third of the esophagus. A few EDCs remain asymptomatic until adulthood, although most EDCs become symptomatic and are discovered in early childhood.^1^, ^2^, ^3^ CT and MRI assist in the preoperative diagnosis of EDCs. Some reports have indicated that endoscopic ultrasonography also plays a crucial role in the diagnosis of EDCs,^2^, ^3^, ^5^ but it was not performed in the present case.

The indications for treating EDCs include symptoms such as dysphagia, aspiration, hemorrhage, an increase in cyst size, and suspicion of malignancy.^1^, ^2^, ^3^, ^4^ Resection of the duplication cyst is the standard treatment, and the approach to the cyst should be determined based on its size, location, and relationship with adjacent organs.^3^, ^4^, ^5^, ^6^ The surgical technique must be based on the integrity of the esophageal mucosa after resection, the condition of the muscular layer over the area where the cyst was present to avoid a pseudodiverticulum, and identification of the vagus nerve. Complete excision of the cyst results in a defect in the muscular layer of the esophagus because of the close relationship between the wall of the cyst and the lumen of the esophagus. The optimal technique for closure of the esophageal muscle layer after EDC resection remains controversial. Benedict et al reported that fewer complications occurred in patients who underwent muscle layer closure during the initial resection and that most patients who required reoperation had their muscle layer left open.^7^ They also reported that more serious postoperative complications such as cyst recurrence, diverticula, strictures, and chylothorax occurred in patients whose muscle layer was left open.^7^ However, a few patients who underwent muscle layer closure developed postoperative GERD despite the absence of preoperative GERD symptoms. Closure of the muscle layer after resection may potentially increase the likelihood of developing GERD, but other possible causes include alteration of the lower esophageal sphincter anatomy or injury to the vagus nerve. Interestingly, two cases in which Nissen fundoplication was applied to laparoscopic resection for an EDC have been reported.^8^, ^9^ The application of Nissen fundoplication might help prevent postoperative complications in patients with EDCs. The Heller–Dor operation is one of the most effective treatments for patients with intractable achalasia, and the surgical procedure involves both Heller myotomy of the esophagus and an anterior 180° Dor fundoplication.^10^ In the present case, we added a Dor fundoplication after tumor resection because we believed that it would potentially prevent postoperative GERD while also reinforcing the exposed muscle layer of the esophagus as effectively as the Heller–Dor operation for achalasia. To our knowledge, this is the first case in which tissue perfusion of the esophagus was assessed using an NIR‐ICG fluorescence system after laparoscopic resection of an EDC. However, further investigations are required because of the rarity of EDCs.

In conclusion, we have described our first case of laparoscopic resection for an EDC using an NIR‐ICG fluorescence system. An NIR‐ICG fluorescence system may be useful for preventing postoperative complications by intraoperatively evaluating tissue perfusion in the region of resection in the esophagus.

## CONFLICT OF INTEREST

The authors have no conflicts of interest to declare.

## AUTHOR CONTRIBUTIONS

M.M., K.S., and A.H. performed the operation and postoperative management. M.M. and K.N. drafted the manuscript, and C.K. and K.K. critically revised the manuscript. All authors read and approved the final manuscript.

## CONSENT FOR PUBLICATION

Written informed consent was obtained from the patient for publication of this case report and any accompanying images. A copy of the written consent is available for review by the Editor‐in‐Chief of this journal.

## References

[1] Kolomainen D , Hurley PR , Ebbs SR . Esophageal duplication cyst: case report and review of the literature. Dis Esophagus. 1998;11(1):62‐65. 9595237

[2] Martin ND, Kim JC , Verma SK , Rubin R , Mitchell DG , Bergin D , Yeo CJ . Intra‐abdominal esophageal duplication cysts: a review. J Gastrointest Surg. 2007;11(6):773‐777. 17562119 10.1007/s11605-007-0108-0

[3] Duan X , Cui Y , He Y , Xu S . Acute attack of recurrent esophageal duplication cyst in an adult: case report and literature review. J Thorac Dis. 2018;10(5):E335‐E339. 29997988 10.21037/jtd.2018.04.89 PMC6006093

[4] Herbella FA , Tedesco P , Muthusamy R , Patti MG . Thoracoscopic resection of esophageal duplication cysts. Dis Esophagus. 2006;19(2):132‐134. 16643183 10.1111/j.1442-2050.2006.00552.x

[5] Joyce AM , Zhang PJ , Kochman ML . Complete endoscopic resection of an esophageal duplication cyst (with video). Gastrointest Endosc. 2006;64(2):288‐289. 16860091 10.1016/j.gie.2006.04.046

[6] Castelijns PS , Woensdregt K , Hoevenaars B , Nieuwenhuijzen GA . Intra‐abdominal esophageal duplication cyst: a case report and review of the literature. World J Gastrointest Surg. 2014;6(6):112‐116. 24976905 10.4240/wjgs.v6.i6.112 PMC4073222

[7] Benedict LA , Bairdain S , Paulus JK , Jackson CC , Chen C , Kelleher C . Esophageal duplication cysts and closure of the muscle layer. J Surg Res. 2016;206(1):231‐234. 27916367 10.1016/j.jss.2016.07.024

[8] Noguchi T , Hashimoto T , Takeno S , Wada S , Tohara K , Uchida Y . Laparoscopic resection of esophageal duplication cyst in an adult. Dis Esophagus. 2003;16(2):148‐150. 12823217 10.1046/j.1442-2050.2003.00314.x

[9] Aldrink JH , Kenney BD . Laparoscopic excision of an esophageal duplication cyst. Surg Laparosc Endosc Percutan Tech. 2011;21(5):e280‐e283. 22002296 10.1097/SLE.0b013e31822f1e67

[10] Patti MG , Pellegrini CA , Horgan S , et al. Minimally invasive surgery for achalasia: an 8‐year experience with 168 patients. Ann Surg. 1999;230(4):587‐593. discussion 593‐594. 10522728 10.1097/00000658-199910000-00014 PMC1420907

